# Comorbidities of Idiopathic Thrombocytopenic Purpura: A Population-Based Study

**DOI:** 10.1155/2009/963506

**Published:** 2009-04-02

**Authors:** M. A. Feudjo-Tepie, G. Le Roux, K. J. Beach, D. Bennett, N. J. Robinson

**Affiliations:** ^1^Worldwide Epidemiology, GlaxoSmithKline R & D, Greenford, Middlesex, UB6 0HE, UK; ^2^Institut National de la Statistique et de l’Analyse Economique (INSAE), Campus de Ker Lann, rue Blaise Pascal, BP 37203, Bruz Cedex 35172, France; ^3^Worldwide Epidemiology, GlaxoSmithKline R & D, Five Moore Drive, Research Triangle Park, NC 27709, USA; ^4^Worldwide Epidemiology, GlaxoSmithKline R & D, Greenford, Middlesex UB6 0HE, UK; ^5^Pharma Development Safety, F. Hoffmann-La Roche Ltd., 4070 Basel, Switzerland

## Abstract

A person experiencing more than one medical condition may have ambiguous clinical presentation. ITP is a serious autoimmune disease with little epidemiological evidence on its burden, risk factors, and comorbidities. Using the United Kingdom general practice research database, we conducted a 14 years population-based case control-type study to explore medical conditions more likely to cooccur with ITP and their temporal relationship in association with ITP. ITP patients were matched to non-ITP on practice, age, gender, and follow-up period. Potential comorbidities were represented by patients' medical information at the preferred term level of the MedDRA international classification. As well as death (OR = 60.0; 95% CI [4.47–806.0]) and known clinical signs and symptoms of ITP, ITP is associated with considerable number of medical conditions. The association between ITP and some of these conditions is apparent both before and after ITP diagnosis. Specific targeted studies can now be setup to reexamine observed associations.

## 1. Introduction

Idiopathic thrombocytopenic purpura (ITP) is a serious acquired autoimmune disorder characterized by a low platelet count (thrombocytopenia) and mucocutaneous bleeding [1]. It is commonly assumed that ITP results from autoantibodies causing accelerated platelet destruction. Recent data suggest that autoantibodies may also inhibit platelet production [2]. The diagnosis of ITP is complex and is very often based on the exclusion of other causes of thrombocytopenia [3–6].

ITP is traditionally divided into acute and chronic forms, based on the duration of thrombocytopenia (i.e., less than 6 months for acute and more than 6 months for chronic) [5]. ITP is generally acute in young children and typically, occurs a few days to a few weeks after an infection (e.g., varicella zoster virus). ITP in children is thought to be a benign and self-limiting disorder with an excellent prognosis. In contrast, ITP in adults is primarily chronic, and the onset is often asymptomatic. The disease is more prevalent in females than males [4].

There is relatively little epidemiological evidence on disease burden and risk factors; prevalence in adults and children may range from 9.6 to 189 per 100 000 person [7–9]. Frederiksen and Schmidt [7] estimated incidence of 2.7 per 100 000 person per year in Denmark. Our recent estimation of the prevalence of chronic ITP in the United State using a large U.S. claim database gave 20.3 per 100 000 person [10].

The coexistence in an individual with two (or more) medical conditions is commonly referred to as comorbidity. More specifically, Feinstein [11] defines comorbidity as “any distinct additional clinical entity, that has existed or that may occur during the clinical course of a patient who has the index disease under study.” A person experiencing more than one condition may present with an ambiguous clinical presentation [12]. Thus, knowledge of disease comorbidities can provide a better understanding of disease burden as well as inform diagnosis and treatment decisions. For example, the investigation of idiopathic normal pressure hydrocephalus (INPH) comorbidities confirmed the early suspicions of INPH as a multi-aetiological clinical entity possibly overlapping physiologically with cerebrovascular and Alzheimer diseases [13]. Disease comorbidities also have consistently been shown to be important prognostic factors of a number of health conditions including cancer, independent of the tumour stage [14–18]. Colinet et al. [19] suggested that comorbidities may explain in part the variability in survival observed from stage I nonsmall cell lung cancer. So, the importance of comorbidity studies is well acknowledged in the literature and their use well documented.

However, evidence on ITP specific comorbidities is sparse. Indeed, apart from the known symptoms of the disease (i.e., bleeding, petechia, purpura, etc), to our knowledge, there is little empirical evidence in the literature on the association, or lack of it, between ITP and other medical conditions. Knowledge of ITP specific comorbidities, that is those frequently occurring with ITP, may provide a better understanding of ITP disease progression, ITP disease burden, and could inform treatment decisions. Their knowledge might lead to preventive measures, early diagnoses, and better disease management. Comorbidities of ITP can also help put into context potential safety signals and improve clinical trial design and planning. Hence, using “real world” evidence, this study aims to reduce the knowledge gap. More specifically, using a large UK healthcare database, the paper describes the basic characteristics of an ITP population and identifies other medical conditions more likely to cooccur or to be diagnosed with ITP. Furthermore, comparing the ITP cohort to a matched non-ITP cohort, the study investigates potential temporal relationship in the association between identified comorbidities and ITP.

## 2. Material and Method

### 2.1. Data Source

The study population includes patients registered on UK's general practice research database (GPRD) during the period 1990–2004. The GPRD has been described elsewhere [20, 21] and has previously been used to study comorbidities of newly diagnosed COPD and asthma [22] among other things. Briefly, the GPRD contains detailed information on diagnoses, prescribing, GP investigations, outcomes, and hospital referrals, together with basic demographic information for about 6 million patients from more than 370 representative general practices throughout the UK. The database is population-based and representative of the age, sex, and geographic regions of the UK [21]. Data quality is monitored continuously by the UK Medicines and Healthcare products Regulatory Agency (MHRA), and practices that fail to maintain the required standards are removed. The completeness and accuracy of the recording of medical information have been validated [23–26]. This study was approved by the scientific ethical and advisory group for the GPRD.

### 2.2. Study Design and Case Definition

A case control study-type approach was utilized. ITP patients (cases) were identified using READ or OXMIS codes (contact authors for details). Patients with less than 1 year of follow-up before or after the first diagnosis of ITP were excluded. For each ITP case up to five, non-ITP patients (controls) were matched on practice, age, gender, and follow-up period. The index date of a case was the first ITP diagnosis date, while that of a control was the index date of his/her matched ITP case. Potential controls also had to have at least one medical record or a recorded prescription in the database within one year before or one year after the ITP diagnosis date.

Potential comorbidities were all medical conditions listed in the GPRD of the cases and/or controls at preferred term level (PTL) of the medical dictionary for regulatory activities (MedDRA) hierarchical classification, over the period of a year before and a year after index date. Additionally, seven selected grouped medical conditions of a priori interest were defined and considered: myocardial infarction, breast cancer, chronic renal failure, hepatitis, liver damage, systemic lupus, and thromboembolic events.

Because the period 1 year before and 1 year after the index date can lead to bias due to differential medical screening between cases and controls [27], the relative frequency of comorbidity between case and controls was further assessed by considering a longer follow-up period. Hence, over the period of 5 years before and 5 years after the index date, trends in risks and risk ratios of identified comorbidities were explored. However, in this latter analysis, in order to reduce the list of considered medical conditions to a manageable size, identified conditions at the preferred term level were grouped in a clinical meaningful way by a clinical epidemiologist with a good knowledge of the UK health system. This pooling of medical conditions into more aggregate groups (hereafter 26 grouped medical conditions) also increases the power of any comparison between cases and controls with respect to the frequency occurrence of any potential comorbidity. Indeed, because in this analysis we were only interested in the first occurrence of potential comorbidities, over the course of the follow-up period, small sample size was an issue. This is the case, particularly for rare conditions.

### 2.3. Statistical Analysis

Age and gender characteristics of ITP patients together with the number of controls per case were described using frequency tables. For each comorbidity, the frequency of its occurrence in cases was compared to that in controls using odds ratios calculated by conditional logistic regression [28]. All medical conditions with odds ratios greater than or equal to 2 and statistically significant at the 0.1% level were selected, ranked by odds ratios and reported. The sensitivity of our chosen significance level of 0.1% to the risk of false positives due to multiple testing was assessed using false discovery rate procedure (FDR) [29].

Trends for each comorbidity over the 10-year period were explored using annual incidence rates (calculated as the number of events over person-years at risk) and rate ratios (with 95% confidence intervals) between ITP and non-ITP patients. Confidence intervals were calculated using the Clayton and Hills formula [30]. To complement the analysis of annual incidence rates, a cumulative risk analysis [31] over the 5-year post index period for each of the comorbidities is performed using log rank test. All analyses are performed using SAS version 9.1.

## 3. Results

### 3.1. Description of Cases and Controls

A total of 1033 incident physician-diagnosed ITP cases during the period 1990–2004 were identified, 578 (56.0%) of whom were females. Table 1 presents the distribution of these patients by age, group, and gender. Approximately, 50% are 50 or over. Mean age was 46 in females and 44 years in males. Following matching, 883 cases (85.5%) had at least one matched control, and these 883 cases were used in further analyses. A total of 3700 controls were selected, and Table 2 presents the distribution of number of controls per case, 69% of cases had 4 or 5 controls.

**Figure 1:**
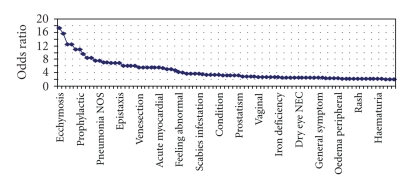
Medical conditions with OR significantly greater than 2 but less than 20.

ITP patients and their matched controls had a total 2366 unique medical conditions at the PTL of the MedDRA classification. Figure 1 and Table 3 show medical conditions with an odds ratio (OR) greater than 2 and significant at the 0.1% level. Conditions are sorted in descending order of OR. Clinical signs or symptoms known to characterize ITP are strongly associated with ITP (i.e., purpura, OR = 146; bruise, OR = 57; neutropenia, OR = 29; etc.). There is a strong association between ITP and death (OR = 60.0; 95% CI [4.47–806.0]). It is also important to note that more than 90 conditions are associated with ITP in this analysis despite our use of stringent criteria (i.e., an odds ratio greater than 2 and statistically significant at a 0.1% level). The use of the false discovery rate procedure to control for the increasing risk of false positives due to multiple testing indicates that setting the significance level of each individual test at 0.35% would have guaranteed a targeted overall 5% level. So, our choice of 0.1% as a significance level is on the cautious side with respect to expected false positives. As noted above, all of these significant conditions were grouped into 26 broader conditions as shown in Table 4.

Some of the medical conditions are rare and/or only found in the ITP group. Because of this, odds ratios, even though quite large (i.e., >2), they may not be statistically significant. Medical conditions with odds ratios greater than 2 but with no more than one event in the control group are thrombocytopenia, petechiae, splenectomy, purpura, haemorrhagic disorder, sepsis, bronchopneumonia, headaches, gingival, guttate psoriasis, acute pancreatitis, pleural effusion, restlessness, and somnolence.

### 3.2. Longitudinal Approach and Cumulative Risk Analysis for Grouped Conditions

Table 5 presents, for each of the 26 grouped medical conditions, over a period of 10 years around the index date, yearly incidence rate ratios, between cases and controls, and their 95% CI. Results can be summarized as follows.

- Conditions that have a significantly higher frequency of occurrence in ITP compared to non-ITP in at least one time period both before and after diagnosis: haematological diseases, dermatological conditions, bleeding disorders, gastrointestinal diseases, autoimmune disorders, and constitution conditions.
- Conditions that have significantly higher frequency of occurrence in ITP compared to non-ITP patients only after index date: oral conditions and infections.
- Conditions that have significantly higher frequency of occurrence in ITP compared to non-ITP patients during only the year of ITP diagnosis or a year before: neurological, coronary artery syndrome/myocardial infarction, genitourinary, musculoskeletal, ophthalmologic, skeletal, and edema.
- Other conditions with no statistical association with ITP.

None of the conditions was found to have a negative association with ITP.

**Figure 2:**
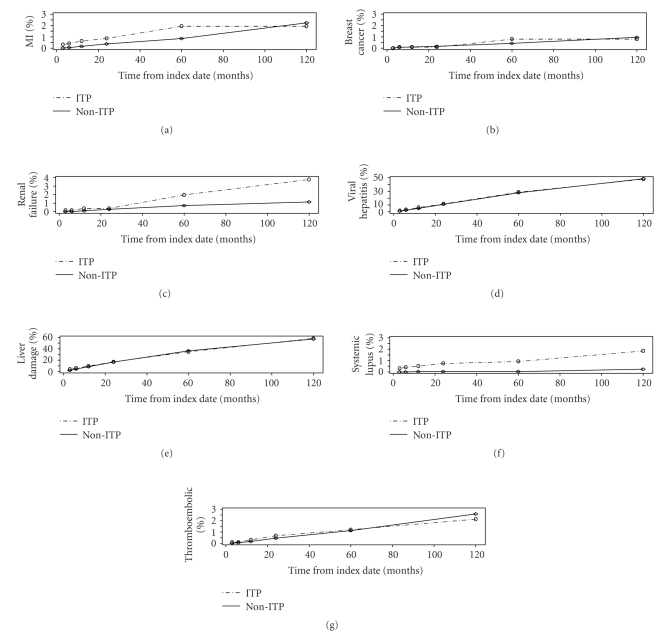
Cumulative risk of 7 grouped conditions over time in ITP and non-ITP populations.

The analysis of conditions of a priori interest (the 7 grouped comorbidities) shows that over the 10-year period, none of the annual incidence rate ratios of the conditions breast cancer, liver disease, viral hepatitis, and thromboembolic events was statistically different from one, at the 5% level (Table 6). This suggests that there is no evidence of an association with ITP. However, there was a suggestion of an association between ITP and myocardial infarction during the last 2 years before ITP diagnosis (RR = 3.63 and RR = 4.69, resp., see Table 6)). There was also evidence of an association between ITP and chronic renal failure during years 4 and 5 after the index date (RR = 6.21 and RR = 6.54, resp.). Although rare (9 total number of observed events), systemic lupus occurred in ITP patients only (i.e., was not observed in control patients), which suggests an association between ITP and systemic lupus (Table 6). Figure 2 shows, for each of the 7 grouped comorbidities, the evolution of their cumulative risk over time (i.e., the overall risk over a specific length of time). Table 7 presents the one- and 5-year risk of all 26 grouped comorbidities together with a log-rank test of difference of occurrence between ITP and non-ITP. These latest analyses further suggest ITP association with medical conditions: neoplasm, constitution, after index date. These associations were not observed in earlier analyses, probably because of insufficient numbers.

## 4. Conclusion/Discussion

We report here an extensive database exploration of medical conditions associated with ITP. First, comparing the frequency of occurrence of each considered disorder in ITP patients and their matched controls, we systematically selected all medical conditions that are likely to be associated with ITP. Then, pooling those selected conditions into more aggregated groups and exploring the trend in their yearly relative frequency over a ten-year period helped us put observed associations into perspective.

We found that ITP is associated with a considerable number of medical conditions, a good number of which to our knowledge were not systematically reviewed before. The association between ITP and some of these conditions (or group of conditions) is apparent both before and after ITP diagnosis. These conditions are hematological diseases, dermatological conditions, bleeding disorders and constitutional conditions, for example, chills, rigors, malaise, and lethargy. The nature of these observed associations suggests that either these conditions share similar causal pathways as ITP or they predispose to the diagnosis of ITP.

There are also conditions associated with ITP only after ITP diagnosis such as oral conditions, infections, gastrointestinal, and autoimmune disorders. This could be indicative of ITP being on the causal pathway of these conditions, an adverse effect of any of the medications generally taken by ITP patients or were conditions not screened for prior to diagnosis of ITP. Further studies are needed here to disentangle the disease (ITP) effect from potential medication effect and other contributing factors.

Conditions associated with ITP only during the years around diagnosis are more likely to be a result of diagnosis bias as mentioned earlier.

Strengths of the current study include design, simplicity, large sample size, and its systematic nature. Indeed, ITP is a relatively rare disease, and only large healthcare databases with long follow-up such as the GPRD could provide an appropriate base population. The design first considers cases and comparable controls and both a cross-sectional and longitudinal approach. By comparing the frequency in ITP patients of potential comorbidities to that in controls matched in practice, gender and length of follow-up and thus, computing the relative risk within the same study base, numbers of confounders are controlled for, including seasonality. We also systematically considered all medical conditions on patients' medical records as potential comorbidities. This is an important consideration for a hypothesis generative study such as this.

A potential limitation of this research is perhaps our subjective grouping of conditions at the preferred term level, in order to obtain aggregated and thus more prevalent groups. We believe, however, that the differences in potential grouping would be unlikely to strongly impact the conclusions. Our attempts at using a more automated and systematic grouping, such as a higher than PTL level of the MedDRA classification, proved difficult, mainly because of the overlapping nature of groups at the higher level of the MedDRA classification.

Children and adults were mixed. Childhood and adulthood ITP may differ significantly, and as comorbidities in the two age groups, it is arguable that a separate analysis of each age group could have added potentially useful information on the age grouping of identified comorbidities. However, we believe that given the reduced number of ITP cases, stratifying our sample into adults and children, there would be less power to detect some of the comorbidities and thus may not be able to identify some of the comorbidities identified when the groups were combined.

Selection bias is also a concern. Within the current design, although the use of a registration period of at least 1 year before any investigation maybe considered standard in database observational research and increases the confidence that new reports of a disease refer to new incident conditions, the requirement of having at least 1 year of enrollment after identification may have biased some estimates. Some patients because of the severity of their condition may have died or been transferred within a year of their diagnosis. The likelihood is that these patients had more comorbidities than those not transferred or who did not die. The impact of this is likely to result in an underestimate of the associations observed. Other sources of bias include differential screening between ITP and non-ITP patients. Patients with a diagnosis of ITP might have a higher chance of receiving a diagnosis of other disease due to regular follow-up GP visits. Thus, the likely relation between GP consultation and frequency of comorbidities imposes cautious interpretation of the extensive list of conditions found here in association with ITP, particularly for those associations only evident during the years around diagnosis date.

Our stringent selection criteria also have limitations in this study. Indeed, we may have missed some of the potential comorbidities because either their OR was less than 2 or the associated *P*-value was greater than 0.1%. Indeed, the false discovery rate indicates that a significant level for each individual test of 0.32% would have guaranteed an overall significance level of 5%. These last two points may explain, for example, why cataracts are not in our list of conditions found in association with ITP. Indeed, oral corticosteroid (OS) use is one of the first line treatments of ITP, and it is expected that a substantial number of our ITP patients would be taking corticosteroids compared with non-ITP patients [5]. A number of studies have consistently shown an increased risk of cataracts associated with corticosteroid use, although the risk is generally not found to be more than two fold [32]. Our stringent criteria excluded comorbidities with less than a two-fold increase in ITP patients compared to non-ITP, as is the case of cataracts.

Another limitation of the current study is our choice of the MedDRA classification. Indeed, data in GPRD are reported by physicians in READ or OXMIS codes and latter mapped to MedDRA. Not all medical conditions would have a correspondent PTL of the MedDRA classification. This may only limits the number of potential comorbidities we could explore. Indeed, there is no reason to believe that conditions with no corresponding PTL on the MedDRA would be systematically more frequent in cases compared to controls. Thus, its impact is more likely to be on increasing the list of potential comorbidities, rather than dismissing some of those selected here.

We did not consider medication use, and thus our analyses are not adjusted for this. Indeed, diseased patients are likely to be taking medications, some of which are associated with a higher risk of other conditions, as it is for cataracts and corticosteroids. Hence, some of the observed associations may be the result of medication use and/or concomitant use of medications and not the result of the condition itself. Also, age influences the pattern of ITP expression [33] through its association with medication use, including for cardiovascular conditions. Hence, some of the identified comorbidities, rather than being directly linked to ITP, may result from a complex interaction between other comorbidities and/or medications used against these comorbidities. Only specific and targeted studies would be able to disentangle the effects of medication, disease, and other factors.

So, to conclude, two or more medical conditions can cooccur because (1) there is a direct causal relationship between these conditions, whereby the presence of one makes the other more likely to develop, (2) there is an indirect causal relationship between the two conditions, whereby one condition affects a third variable in a way that makes the second condition more likely to develop, (3) there are common factors that increase the risk of both disorders, (4) the occurrence of one of the medical conditions is associated with medication used to treat the other, or (5) other reasons [34]. The diagnosis of ITP is complex and based on exclusion of other causes of thrombocytopenia. There is also relatively little epidemiological evidence on the disease. To the best of our knowledge, this is the first study using large healthcare databases to systematically explore comorbidities of ITP. This study aimed to support what we know and then potentially glean additional insights from this rich data source, which might then trigger further interest in setting up additional specific focused studies. This should be done in a variety of settings. These targeted studies should also help the understanding of the underlying mechanism. The current exploration suggests that ITP, despite being a rare disease, is associated with a considerably higher level of mortality risk as well as an extensive list of comorbid medical conditions. For ITP patients, this increased quantitative understanding of ITP comorbidities hopefully may assist in improved disease management of ITP patients.

## Figures and Tables

**Table 1:** ITP patients by age and gender.

Age (years)	Gender		Total N (%*)
	Female *n* (%*)	Male *n* (%*)	
0–15	94 (16.3)	131 (28.8)	225 (21.7)
16–49	208 (36.0)	95 (20.9)	303 (29.3)
50 +	276 (47.7)	229 (50.3)	505 (49.9)
Total	578 (100.0)	455 (100.0)	1033 (100.0)

*Proportions are column proportions.

**Table 2:** Distribution of number of controls per case.

Number of controls per case	Frequency (cases)	Percent	Cumulative percent
0	150	14.5	14.52
1	64	6.20	20.72
2	39	3.78	24.49
3	68	6.58	31.07
4	206	19.9	51.02
5	506	49.0	100.00

**Table 3:** Comorbidities significantly associated with ITP in the period or 2 years around diagnosis date.

*P*	Odds ratio	CL		Preferred term name	*P*	Odds ratio	CL		Preferred term name
<.0001	146.00	23.48	907.97	Purpura	.0009	3.30	1.30	8.36	Breast cancer female NOS
<.0001	146.00	23.48	907.97	Purpura NOS	.0009	3.30	1.30	8.36	Condition aggravated
<.0001	60.00	4.47	805.56	Death	.0008	3.18	1.31	7.75	Haemoptysis
<.0001	60.00	4.47	805.56	Death NOS	.0008	3.18	1.31	7.75	Confusion
<.0001	57.33	19.85	165.60	Increased tendency to bruise	<.0001	3.17	1.58	6.35	Diabetes mellitus noninsulin-dependent
.0009	29.00	2.11	398.24	Neutropenia	<.0001	3.17	1.58	6.35	Diabetes mellitus noninsulin-dependent
<.0001	21.50	3.34	138.56	Whole blood transfusion	<.0001	3.14	1.65	5.99	Prostatism
<.0001	17.31	10.84	27.65	Ecchymosis	.0001	2.94	1.43	6.06	Left ventricular failure
<.0001	15.63	9.97	24.49	Contusion	.0008	2.92	1.28	6.69	Respiratory disorder
.0006	12.50	1.88	82.96	Chills	.0008	2.92	1.28	6.69	Respiratory disorder NOS
.0006	12.50	1.88	82.96	Rigors	.0004	2.81	1.33	5.95	Analgesic effect
<.0001	11.00	2.33	51.99	Chemoprophylaxis NOS	<.0001	2.73	1.51	4.93	Vaginal haemorrhage
<.0001	11.00	2.33	51.99	Prophylactic chemotherapy	.0005	2.71	1.30	5.62	Dermatitis diaper
<.0001	9.50	2.45	36.79	Haematemesis	.0007	2.69	1.26	5.71	Phlebitis NOS
<.0001	8.43	4.07	17.46	White blood cell disorder	<.0001	2.67	1.44	4.94	Oral candidiasis
<.0001	8.43	4.07	17.46	White blood cell disorder NOS	.0002	2.65	1.35	5.21	Postoperative analgesia
<.0001	7.57	2.69	21.33	Pneumonia	<.0001	2.61	1.54	4.42	Iron deficiency anaemia
<.0001	7.57	2.69	21.33	Pneumonia NOS	<.0001	2.59	1.58	4.27	Anaemia
<.0001	7.00	2.04	23.98	Blister	<.0001	2.59	1.58	4.27	Anaemia NOS
<.0001	7.00	1.77	27.74	Dry mouth	<.0001	2.59	1.45	4.65	Osteoporosis
<.0001	6.90	2.89	16.50	Leg ulcer (exc varicose)	<.0001	2.59	1.45	4.65	Osteoporosis NOS
<.0001	6.90	2.89	16.50	Leg ulcer (excl varicose)	<.0001	2.58	1.42	4.67	Dry eye NEC
<.0001	6.82	4.05	11.49	Epistaxis	<.0001	2.58	1.42	4.67	Dry eye NOS
.0009	6.00	1.49	24.12	Dementia	<.0001	2.57	1.45	4.56	Keratoconjunctivitis sicca
.0009	6.00	1.49	24.12	Dementia NOS	.0005	2.50	1.26	4.94	Lethargy
.0009	6.00	1.49	24.12	Decubitus ulcer	<.0001	2.44	1.47	4.07	General symptom
.0009	6.00	1.49	24.12	Pressure sore	<.0001	2.44	1.47	4.07	General symptom NOS
.0004	5.60	1.60	19.56	Venesection	<.0001	2.44	1.53	3.89	Rectal haemorrhage
.0004	5.60	1.60	19.56	Venipuncture	.0004	2.37	1.27	4.44	Mouth ulceration
<.0001	5.55	2.39	12.89	Peripheral swelling	.0004	2.31	1.26	4.23	Cardiac failure congestive
<.0001	5.53	2.80	10.90	Eczema gravitational	.0008	2.30	1.21	4.38	Cerebrovascular accident
<.0001	5.53	2.80	10.90	Stasis dermatitis	<.0001	2.27	1.60	3.22	Oedema peripheral
<.0001	5.50	2.27	13.33	Acute myocardial infarction	.0008	2.24	1.21	4.16	Gastro-oesophageal reflux disease
.0005	5.40	1.54	18.92	Intermittent claudication	.0008	2.24	1.21	4.16	Gastro-oesophageal reflux disease
<.0001	5.00	1.95	12.80	Abdominal pain lower	<.0001	2.21	1.58	3.10	Viral infection
.0003	5.00	1.58	15.82	Hip arthroplasty	<.0001	2.21	1.58	3.10	Viral infection NOS
<.0001	4.71	2.21	10.06	Myocardial infarction	<.0001	2.21	1.70	2.87	Rash
<.0001	4.24	2.39	7.52	Feeling abnormal	<.0001	2.21	1.70	2.87	Rash NOS
<.0001	3.95	2.11	7.41	Skin ulcer	<.0001	2.15	1.31	3.52	Menorrhagia
<0.0001	3.71	1.71	8.07	Dry eye	<.0001	2.13	1.38	3.29	Diabetes mellitus
.0006	3.67	1.39	9.66	Folliculitis	<.0001	2.13	1.38	3.29	Diabetes mellitus NOS
<.0001	3.64	1.67	7.92	Acarodermatitis	.0004	2.12	1.23	3.65	Haematuria
<.0001	3.64	1.67	7.92	Scabies infestation	<0.0001	2.12	1.36	3.30	Haemorrhoids
<.0001	3.60	1.43	9.04	Angina unstable	<.0001	2.08	1.33	3.26	Prophylaxis
<.0001	3.35	2.23	5.03	Malaise	<.0001	2.08	1.33	3.26	Prophylaxis NOS
.0009	3.30	1.30	8.36	Oral pain	.0002	2.02	1.24	3.29	Atrial fibrillation

**Table 4:** Comorbidities at the PTL of the MedDRA classification and their 26 grouping.

Grouped conditions	Medical conditions at preferred term level
Bleeding	Ecchymosis, haemoptysis, conjunctival haemorrhage, menorrhagia, rectal haemorrhage, gastrointestinal haemorrhage NOS*, haematuria, epistaxis, haematemesis
Pulmonary	Respiratory disorder, respiratory disorder NOS*
Constitution	Chills, rigors, malaise, general symptom, feeling abnormal, lethargy
Peripheral vascular diseases	Phlebitis NOS, aortic aneurysm, phlebitis, intermittent claudication
Cerebrovascular *accident*	Cerebrovascular accident
Haematology	White blood cell disorder, white blood cell disorder NOS, neutropenia, anaemia, iron deficiency anaemia, anaemia NOS
*Arrhythmia*	Atrial fibrillation
*Autoimmune*	Systemic lupus erythematosus, rheumatoid arthritis
*Renal*	Renal failure acute
*Infection*	Viral infection, pneumonia, viral infection NOS, pneumonia NOS
*Oral conditions*	Oral candidiasis, dry mouth, mouth ulceration, oral pain
*Hypoglycemia*	Hypoglycaemia, hypoglycaemia NOS
*Neoplasm malignant*	Neoplasm malignant
*Dermatological conditions*	Leg ulcer (exc varicose), rash scaly, skin exfoliation, eczema gravitational, stasis dermatitis, decubitus ulcer, pressure sore, folliculitis, skin ulcer, tinea NOS, rash, infected sebaceous cyst, dermatitis diaper, rash NOS
*Hypersensitivity*	Hypersensitivity, hypersensitivity NOS
*Breast cancer*	Breast cancer female NOS, breast cancer, breast cancer NOS
*Neurology*	Confusional state, dementia, dementia NOS, confusion
*Diabetes*	Diabetes mellitus noninsulin-dependent, diabetes mellitus noninsulin-dependent, diabetes mellitus, diabetes mellitus NOS
*Edema*	Peripheral swelling, oedema peripheral
*Genitourinary*	Prostatism, proctalgia
Coronary artery syndrome/myocardial infarction	Acute myocardial infarction, myocardial infarction, angina unstable
*Congestive heart failure*	Left ventricular failure, cardiac failure congestive
*Gastro intestinal*	Haemorrhoids, abdominal pain lower, gastro-oesophageal reflux disease, gastro-oesophageal reflux disease, colitis, colitis NOS
*Ophthalmology*	Dry eye NEC, dry eye, eyelid ptosis, eye pain, dry eye NOS, keratoconjunctivitis sicca
*Musculo-skeletal conditions*	Osteoporosis, hip arthroplasty, ligament sprain, musculoskeletal pain, osteoporosis NOS
*Mood disorder*	Dysthymic disorder

*Not otherwise specified.

**(a):** 

Comorbidity group	Ratio ITP/non-ITP														
	[−5 −4[			[−4 −3[			[3 −2[			[−2 −1[			[−1 diag[		

Rate ratio	Lower CI	Upper CI	Rate ratio	Lower CI	Upper CI	Rate ratio	Lower CI	Upper CI	Rate ratio	Lower CI	Upper CI	Rate ratio	Lower CI	Upper CI
CASMI	1.4	0.3	7.4	0.9	0.2	4.3	2.7	0.9	7.8	**3.1**	1.0	9.3	**4.0**	1.7	9.5
PVD	1.7	0.6	4.8	1.1	0.3	4.0	3.0	0.9	10.0	**4.6**	1.8	11.6	1.5	0.5	4.4
Heme	**2.8**	1.3	5.7	**2.2**	1.0	5.0	**3.0**	1.4	6.2	**2.1**	1.1	4.0	**4.9**	3.2	7.6
Renal	NA	—	—	0.0	—	—	NA	—	—	3.6	0.2	57.3	7.2	0.7	79.1
Pulmonary	12	0.1	11.5	0.0	—	—	7.2	0.7	79.1	**4.8**	1.1	21.4	1.2	0.3	4.4
Oral	1.4	0.7	2.7	1.5	0.7	3.2	0.9	0.4	2.2	1.1	0.6	2.2	**2.8**	1.4	5.3
Ophthalmologic	2.3	0.9	5.9	1.4	0.5	3.9	1.3	0.5	3.2	1.5	0.7	3.5	**2.6**	1.2	5.4
Neurological	0.0	—	—	0.7	0.1	6.1	3.6	0.5	25.4	**4.8**	1.1	21.4	**7.2**	1.8	28.8
Neoplasm	3.6	0.2	57.4	1.8	0.2	19.8	NA	—	—	0.0	—	—	0.7	0.1	6.1
Musculo-skeletal	1.6	0.7	3.7	1.0	0.3	3.1	1.3	0.6	3.2	1.4	0.5	3.5	**2.6**	1.3	5.3
Mood disorder	1.8	0.4	7.2	0.0	—	—	3.6	0.2	57.3	0.0	—	—	Inf	—	—
Infections	1.3	0.6	2.8	1.3	0.6	2.8	1.4	0.6	2.9	1.4	0.8	2.5	**2.9**	1.8	4.6
Hypoglycemia	0.0	—	—	NA	—	—	Inf	—	—	0.0	—	—	3.6	0.2	57.3
Hypersensitivity	**4.8**	1.1	21.4	1.4	0.3	7.4	2.7	0.6	12.1	0.0	—	—	0.7	0.2	3.3
GU	3.6	0.7	17.8	1.2	0.3	4.4	2.1	0.6	7.0	0.4	0.1	2.0	1.0	0.3	3.1
GI	0.9	0.4	2.3	1.4	0.7	2.7	0.9	0.4	2.0	**1.8**	1.0	3.0	**2.4**	1.5	3.9
Edema	1.6	0.6	3.8	1.5	0.6	3.6	0.6	0.2	1.7	1.8	0.8	3.8	1.5	0.8	2.7
Diabetes	1.4	0.5	3.9	0.9	0.3	2.5	1.8	0.6	5.3	1.5	0.6	3.3	1.6	0.7	3.4
Derm	1.1	0.6	1.9	**1.5**	1.0	2.3	1.2	0.8	1.9	**1.6**	1.0	2.3	**2.5**	1.9	3.3
CV	0.0	—	—	3.6	0.5	25.4	2.2	0.5	9.0	1.8	0.3	9.8	**4.3**	1.3	14.1
Constitution	1.4	0.6	3.1	1.3	0.7	2.5	**2.3**	1.2	4.2	**1.7**	1.0	3.0	**2.8**	1.8	4.6
CHF	0.0	—	—	2.2	0.5	9.0	1.1	0.3	3.9	2.7	0.9	7.8	1.0	0.3	3.1
Breast cancer	3.6	0.2	57.1	0.9	0.1	8.0	NA	—	—	1.8	0.3	9.8	1.8	0.3	9.8
Bleeding	1.3	0.8	2.1	**1.6**	1.0	2.7	**1.8**	1.2	2.8	**2.2**	1.5	3.3	**9.0**	6.8	11.9
Autoimmune	3.6	0.7	17.9	5.4	0.9	32.6	Inf	—	—	1.2	0.1	11.7	**6.1**	1.5	25.4
Arrhythmia	0.7	0.2	3.3	0.0	—	—	0.3	0.0	2.0	0.7	0.2	3.3	**3.6**	1.4	9.1

**(b):** 

Comorbidity group	Ratio ITP/non-ITP															
	[diag +1[			[+1 +2[			[+2 +3[			[+3 +4[			[+4 +5[		

Rate ratio	Lower CI	Upper CI	Rate ratio	Lower CI	Upper CI	Rate ratio	Lower CI	Upper CI	Rate ratio	Lower CI	Upper CI	Rate ratio	Lower CI	Upper CI	Ntotal ITP/ year
CASMI	1.9	0.7	5.6	0.9	0.2	4.1	2.0	0.5	8.0	1.0	0.1	9.3	4.4	0.9	21.6	4.1
PVD	**3.4**	1.2	9.4	0.5	0.1	2.4	2.4	0.9	6.7	1.7	0.3	8.7	1.8	0.3	9.2	4.7
Heme	**3.8**	2.2	6.6	**2.4**	1.3	4.5	**5.9**	3.0	11.6	2.0	0.8	5.2	0.8	0.2	2.7	16.4
Renal	Inf	—	—	Inf	—	—	0.0	—	—	0.0	—	—	4.3	0.3	68.8	0.7
Pulmonary	**3.8**	0.9	15.1	2.5	0.8	7.6	0.8	0.1	6.8	0.8	0.1	7.1	0.7	0.1	6.0	2.2
Oral	**3.6**	1.9	6.8	**3.1**	1.5	6.4	0.5	0.1	2.2	1.8	0.6	5.0	1.0	0.3	3.6	9.5
Ophthalmologic	**2.8**	1.4	5.7	0.9	0.3	2.8	1.1	0.3	3.9	0.9	0.3	3.1	1.3	0.4	4.1	6.5
Neurological	2.4	0.9	6.2	0.2	0.0	1.7	1.8	0.5	5.7	2.7	0.8	9.7	0.6	0.1	5.0	3.0
Neoplasm	Inf	—	—	0.0	—	—	8.0	0.7	87.8	0.0	—	—	0.0	—	—	1.0
Musculo-skeletal	**3.8**	2.1	7.0	1.6	0.6	4.2	1.4	0.4	4.2	1.7	0.7	4.4	0.8	0.2	3.7	7.7
Mood disorder	1.9	0.2	20.7	0.0	—	—	0.0	—	—	4.1	0.3	64.9	Inf	—	—	0.8
Infections	**1.9**	1.2	3.1	1.7	0.9	3.0	**2.2**	1.1	4.2	2.0	0.8	4.5	0.5	0.1	2.0	13.5
Hypoglycemia	5.7	0.9	33.8	2.0	0.2	21.8	2.6	0.4	15.8	0.0	—	—	NA	—	—	0.8
Hypersensitivity	**4.3**	1.6	11.9	2.0	0.5	7.9	0.7	0.1	3.0	0.0	—	—	0.0	—	—	2.4
GU	**3.5**	1.5	7.9	2.2	0.8	5.9	0.7	0.1	3.0	1.4	0.4	5.1	2.9	0.5	17.3	4.0
GI	**2.0**	1.1	3.8	1.2	0.6	2.2	**1.9**	1.0	3.7	0.7	0.3	1.8	**2.7**	1.2	6.1	12.7
Edema	**2.4**	1.4	3.9	0.6	0.2	1.6	0.2	0.1	0.9	0.9	0.3	2.3	0.7	0.2	2.3	8.4
Diabetes	2.1	0.8	5.2	1.0	0.3	3.0	1.2	0.3	4.3	1.2	0.2	5.7	0.7	0.1	5.9	4.8
Derm	**2.5**	1.8	3.6	1.0	0.7	1.6	**1.6**	1.0	2.6	1.3	0.8	2.4	0.9	0.4	1.9	30.1
CV	1.9	0.7	5.0	0.7	0.1	2.9	2.2	0.7	6.6	0.0	—	—	1.2	0.3	6.0	2.8
Constitution	**2.3**	1.6	3.5	1.1	0.7	1.9	**1.8**	1.0	3.3	1.6	0.8	3.1	1.2	0.5	3.1	17.6
CHF	1.5	0.7	3.4	0.7	0.1	2.9	0.9	0.2	4.1	0.4	0.1	3.2	0.6	0.1	4.9	3.0
Breast cancer	0.0	—	—	0.0	—	—	0.0	—	—	Inf	—	—	0.0	—	—	0.8
Bleeding	**2.9**	2.0	4.1	**3.9**	2.6	5.7	**1.7**	1.0	3.0	0.7	0.3	1.7	**2.3**	1.3	4.2	40.1
Autoimmune	**15.4**	1.7	137.5	2.0	0.4	11.0	**12.2**	1.3	117.2	2.1	0.2	23.3	0.0	—	—	2.3
Arrhythmia	1.6	0.7	3.6	0.0	—	—	1.6	0.3	8.1	0.2	0.0	1.6	0.0	—	—	2.5

**(a):** 

Comorbidity group	Ratio ITP/non-ITP														
	[−5 −4[			[−4 −3[			[3 −2[			[−2 −1[			[−1 diag[		

Rate ratio	Lower CI	Upper CI	Rate ratio	Lower CI	Upper CI	Rate ratio	Lower CI	Upper CI	Rate ratio	Lower CI	Upper CI	Rate ratio	Lower CI	Upper CI
MI	0.9	0.10	8.10	0.6	0.07	5.0	2.6	0.8	8.1	**3.6**	1.0	12.5	**4.7**	1.7	12.6
Breast cancer	1.8	0.2	19.7	0.9	0.1	8.0	0.0	—	—	**1.4**	0.3	7.3	2.1	0.5	8.9
Chronic renal failure	**1.8**	0.3	9.8	**1.2**	0.12	11.5	**0.9**	0.1	8.0	**2.7**	0.6	12.1	**2.6**	0.8	8.1
Viral hepatitis	1.2	0.7	2.2	1.5	0.8	2.6	0.9	0.5	1.5	0.7	0.4	1.4	0.7	0.4	1.25
Liver damage	—	—	—	—	—	—	—	—	—	—	—	—	—	—	—
Systemic lupus	1.8	0.2	19.8	NA	—	—	3.6	0.2	57.4	NA	—	—	**Inf**	—	—
Thrombo-embolic	1.2	0.1	11.5	0.9	0.1	8.1	1.03	0.21	4.9	7.2	1.3	39.4	1.4	0.4	5.1

**(b):** 

Comorbidity group	Ratio ITP/non-ITP														
	[diag +1[			[+1 +2[			[+2 +3[			[+3 +4[			[+4 +5[		

Rate ratio	Lower CI	Upper CI	Rate ratio	Lower CI	Upper CI	Rate ratio	Lower CI	Upper CI	Rate ratio	Lower CI	Upper CI	Rate ratio	Lower CI	Upper CI
MI	3.18	0.97	10.4	1.14	0.2	5.5	2.4	0.6	10.1	1.0	0.1	9.3	4.4	0.9	21.6
Breast cancer	**0.9**	0.1	8.4	0.0	—	—	0.0	—	—	inf	—	—	2.15	0.2	23.7
Chronic renal failure	**3.0**	0.8	11.3	**0.0**	—	—	**1.9**	0.5	7.9	6.2	1.04	37.1	6.5	1.1	39.1
Viral hepatitis	0.6	0.3	1.2	0.8	0.4	1.6	0.7	0.3	1.7	1.2	0.6	2.3	1.3	0.5	3.2
Liver damage	—	—	—	—	—	—	—	—	—	—	—	—	—	—	—
Systemic lupus	**Inf**	—	—	**Inf**	—	—	NA	—	—	NA	—	—	**Inf**	—	—
Thrombo-embolic	1.3	0.3	6.3	1.2	0.3	4.3	0.0	—	—	0.8	0.1	7.1	1.7	0.3	8.9

**Table 7:** One- and five-year risk of 26-grouped comorbidities and tests over strata after index.

Comorbidities grouped	Test over strata ITP/non-ITP	ITP cohort *n* = 883				Non-ITP cohort *n* = 3700			
	log-rank*	N	Nb comor b	1-year risk	5-year risk	N	Nb comor b	1-year risk	5-year risk
Coronary artery syndrome/myocardial infarction	NS	982	15	0.63%	1.95%	3601	47	0.25%	1.12%
Peripheral vascular diseases	NS	976	22	0.85%	2.86%	3598	60	0.19%	1.45%
Haematological conditions	S	912	90	0.00%	12.59%	3478	149	0.00%	4.37%
Autoimmune	S	1004	13	0.50%	1.39%	3662	14	0.03%	0.28%
Renal	NS	1030	4	0.20%	0.58%	3696	5	0.00%	0.08%
Pulmonary	NS	1022	16	0.42%	1.58%	3678	45	0.11%	1.06%
Oral	S	953	51	2.06%	5.76%	3468	122	0.61%	2.98%
Ophthalmologic	NS	974	32	1.40%	3.81%	3546	97	0.42%	2.88%
Neurological	NS	1021	24	0.80%	2.53%	3682	72	0.27%	1.82%
Neoplasm	S	1026	7	0.51%	0.76%	3685	10	0.00%	0.31%
Musculo-skeletal	S	974	51	2.04%	5.17%	3540	113	0.59%	2.78%
Mood disorder	NS	1021	4	0.10%	0.29%	3661	13	0.05%	0.28%
Infections	S	946	77	2.52%	8.78%	3503	203	1.37%	5.58%
Hypoglycemia	NS	1030	8	0.30%	0.69%	3696	15	0.05%	0.33%
Hypersensitivity	NS	1008	18	0.00%	2.13%	3630	49	0.00%	1.77%
Genitrourinary	S	1000	28	1.03%	3.19%	3597	59	0.31%	1.65%
GastroIntestimnal	NS	907	64	1.96%	8.76%	3324	216	0.81%	5.52%
Edema	NS	970	53	2.58%	5.24%	3508	198	1.08%	5.50%
Diabetes	NS	972	21	0.74%	2.26%	3546	68	0.33%	2.89%
Dermatological conditions	S	788	158	7.19%	20.66%	3107	493	2.67%	13.87%
Cerebrovascular conditions	NS	1015	18	0.62%	2.00%	3661	56	0.30%	1.53%
Constitution	S	916	109	0.00%	14.98%	3425	299	0.00%	9.50%
Congestive heart failure	NS	1010	17	0.81%	1.82%	3636	70	0.52%	2.05%
Breast cancer	NS	1025	3	0.00%	0.30%	3670	15	0.00%	0.44%
Bleeding	S	671	167	9.23%	27.82%	3059	498	2.55%	11.91%
Arrhythmia	NS	1006	14	0.82%	2.32%	3629	81	0.52%	2.32%

*NS = Not significant at 5% level; S = Significant at 5% level.
